# Tetramer-Based Enrichment of Preexisting Anti-AAV8 CD8^+^ T Cells in Human Donors Allows the Detection of a T_EMRA_ Subpopulation

**DOI:** 10.3389/fimmu.2019.03110

**Published:** 2020-01-21

**Authors:** Céline Vandamme, Rebecca Xicluna, Leslie Hesnard, Marie Devaux, Nicolas Jaulin, Mickaël Guilbaud, Johanne Le Duff, Célia Couzinié, Philippe Moullier, Xavier Saulquin, Oumeya Adjali

**Affiliations:** ^1^INSERM UMR 1089, Université de Nantes, CHU de Nantes, Nantes, France; ^2^CRCINA, INSERM, CNRS, Université d'Angers, Université de Nantes, Nantes, France

**Keywords:** AAV, gene therapy, immune response, CD8^+^ T lymphocytes, tetramer-associated magnetic enrichment

## Abstract

Pre-existing immunity to AAV capsid may compromise the safety and efficiency of rAAV-mediated gene transfer in patients. Anti-capsid cytotoxic immune responses have proven to be a challenge to characterize because of the scarcity of circulating AAV-specific CD8^+^ T lymphocytes which can seldom be detected with conventional flow cytometry or ELISpot assays. Here, we used fluorescent MHC class I tetramers combined with magnetic enrichment to detect and phenotype AAV8-specific CD8^+^ T cells in human PBMCs without prior amplification. We showed that all healthy individuals tested carried a pool of AAV8-specific CD8^+^ T cells with a CD45RA^+^ CCR7^−^ terminally-differentiated effector memory cell (T_EMRA_) fraction. *Ex vivo* frequencies of total AAV-specific CD8^+^ T cells were not predictive of IFNγ ELISpot responses but interestingly we evidenced a correlation between the proportion of T_EMRA_ cells and IFNγ ELISpot positive responses. T_EMRA_ cells may then play a role in recombinant AAV-mediated cytotoxicity in patients with preexisting immunity. Overall, our results encourage the development of new methods combining increased detection sensitivity of AAV-specific T cells and their poly-functional assessment to better characterize and monitor AAV capsid-specific cellular immune responses in the perspective of rAAV-mediated clinical trials.

## Introduction

Over the past decade, recombinant adeno-associated virus-derived vectors (rAAV) have emerged as a powerful vector platform for *in vivo* gene delivery. With over a 100 gene therapy clinical trials worldwide, sustained therapeutic effect has been achieved in the frame of a variety of inherited diseases such as Leber's congenital amaurosis type 2 (1, 2), hemophilia B (3), M-type α-1 antitrypsin deficiency (4), or lipoprotein lipase deficiency (5). Already three different AAV-based gene therapy products have received market approval [Glybera (6), Luxturna (7), Zolgensma (8)]. Nevertheless, all these successes have been tempered by rising concerns over the immunogenicity of the AAV capsid in patients, especially when the vector was delivered *via* a systemic route.

Adeno-Associated Viruses (AAV) are small, non-enveloped, DNA dependo-viruses belonging to the *Parvoviridae* family. Though widely disseminated among the human population (6), wild-type (WT) AAV human infection has not been clearly associated to clinical outcome. Seroprevalence studies have indicated that initial exposure to WT AAV often occurs early during childhood (7, 8), when humoral and cellular immune responses directed against the AAV capsid might be mounted (9, 10). As such, memory AAV-specific T and B cells might be retained throughout lifetime and recalled upon rAAV-mediated gene transfer. While the prevalence of anti-AAV antibodies among the human population is widely studied today (11), and their impact on rAAV-mediated gene transfer is fairly well-documented (12), the detection and characterization of AAV-specific T cell responses remain somewhat more of a challenge even if this issue was first addressed more than 15 years ago (13).

Deleterious effects of anti-AAV cellular immune responses were first evidenced in a liver-directed gene transfer clinical trial for severe hemophilia B patients, where an AAV serotype 2 (AAV2) vector carrying the coagulation factor IX transgene was administered *via* the intrahepatic route (9). In this study, gradual loss of factor IX transgene expression correlated with transient rise in liver transaminase levels and increase in the frequency of AAV-specific CD8^+^ T lymphocytes (10). Those observations were further confirmed in the same clinical indication when the AAV8 serotype was administered intravenously (11). Tremendous amount of work has been done to understand the underlying mechanisms of AAV capsid-specific CD8^+^ T cell cytotoxicity. The current working model states that upon rAAV administration, transduced hepatocyte cells are able to process, and present capsid-derived epitopes onto major histocompatibility class I (MHC I) molecules. Those peptide-MHC (p-MHC) complexes serve as docking sites for recognition by memory capsid-specific CD8^+^ T cells which then activate and expand, leading to the destruction of the transduced cells (12). Notwithstanding, it is still currently impossible to predict the onset of AAV-specific CD8^+^ T cell responses in patients and their clinical impact as positive ELISpot responses don't always correlate with loss of transgene expression (3). One can put forward three main reasons for these limitations: (1) The absence of a relevant animal model recapitulating what is observed in patients; (2) An outcome shown to be variable between individuals and potentially dependent on the target tissue (i.e., liver vs. skeletal muscle) and route of rAAV delivery; and more importantly; (3) The difficulty to monitor AAV-specific CD8^+^ T cells *ex vivo* without prior amplification of PBMCs or splenocytes because of their scarcity leading to a lack of data on their phenotype and functionality.

As recent technological breakthroughs now allow direct *ex vivo* assessment of even scarce antigen-specific CD8^+^ T cell populations, we first addressed the issue of detecting low capsid-specific CD8^+^ T cell frequencies. We applied a p-MHC tetramer-based enrichment approach (later referred to as TAME, for Tetramer-Associated Magnetic Enrichment) (13, 14), with a flow cytometry-based read out, to analyze the presence and frequency of AAV- specific CD8^+^ T cells within the peripheral blood mononuclear cells (PBMCs) of healthy donors. We were able to detect AAV- specific CD8^+^ T cells in a large cohort of donors and we further identified among them a subset of CD45RA^+^ CCR7^−^ terminally-differentiated effector memory cells (T_EMRA_). After flow cytometric cell sorting we generated primary AAV8-specific CD8^+^ T cell lines that were able to secrete IFNγ, TNFα as well as mediate cytotoxicity toward capsid-loaded targets *in vitro*. Importantly, there was no correlation between anti-AAV IFNγ ELISpot responses and *ex vivo* frequency of total AAV8-specific CD8^+^ T cells, suggesting that frequency of capsid-reactive T cells is not predictive of their functionality. However, we found a correlation between T_EMRA_ frequencies and IFNγ ELISPOT positive responses indicating that T_EMRA_ cells may play a role in recombinant AAV-mediated cytotoxicity in patients with preexisting immunity. In conclusion, our data emphasize the development of new methods combining increased detection sensitivity of AAV-specific T cells and their poly-functional assessment, to better understand capsid-specific cellular immune responses and ultimately predict their impact in rAAV-mediated clinical trials.

## Materials and Methods

All this work was performed under the control of our quality management system that is approved by Lloyd's Register Quality Assurance LRQA to meet requirements of international Management System Standards ISO 9001:2015. It has been implemented to cover all activities in the laboratory, including research experiments and production of research-grade viral vectors.

### Vector Production

For ELISA and neutralization assays, single-stranded AAV serotypes 2 and 8 vectors were produced by the vector core facility in Nantes (https://umr1089.univ-nantes.fr/plateaux-technologiques/cpv/). Vectors were produced through co-transfection of Human Embryonic Kidney 293 cells, purified by cesium-chloride gradient, and titrated by dot plot and quantitative PCR assays. For rAAV vectors used in neutralization assays, the vector plasmid contained the LacZ reporter transgene.

### Donor Samples

Cytapheresis samples were provided by the local Etablissement Français du Sang (EFS Nantes, Pays de la Loire, agreement N° PLER NTS 2016-25) and originated from consenting healthy donors living in the surrounding area (4 women, 61 men). Median age was 46.5 years old (min: 20; max: 72). Plasma were obtained by centrifuging undiluted cytapheresis samples, and were stored at −80°C. PBMCs were obtained by Ficoll density gradient centrifugation (Ficoll-Paque PLUS, GE Healthcare). A fraction of PBMCs was frozen in liquid nitrogen for ELISpot assays, while remaining fresh PBMCs were used for tetramer-based enrichment. HLA-A2 or HLA-B7 phenotyping of PBMCs was performed by flow cytometry using FITC-conjugated anti-HLA-A2 (clone BB7.2, BD Biosciences) or FITC-conjugated anti-HLA-B7 (clone BB7.1, Bio-Rad) antibodies.

### Peptides and Peptide/MHC (pMHC) Tetramer Complexes

HLA-A2- and HLA-B7-restricted peptides were purchased from GL Biochem (Singapore) (Table S1). Immunodominant peptides (LIDQYLYYL and IPQYGYLTL) were provided by Proteogenix (France). Biotinylated A2- or B7-soluble MHC class I monomers loaded with conditional ligands (15) were produced by the Plateforme de Protéines Recombinantes (P2R, Nantes, France). Biotinylated A2- or B7- soluble monomers loaded with AAV2 or AAV8 capsid-derived peptides were obtained through UV-mediated peptide exchange as previously described (15). Oligomerization of pMHC monomers was performed with PE- or APC-labeled tetramer-grade streptavidin (PJRS27, Prozyme, and BD Biosciences *resp*.) during 1 h at 4°C, at a molar ratio of 4:1, to form low-order oligomers referred to as “tetramers.”

### Tetramer-Based Enrichment Protocol

2 × 10^8^ freshly isolated PBMCs were incubated with a mix containing 100 μL of AAV capsid-specific PE-conjugated tetramers (20 μg/mL) and 100 μL of irrelevant APC-conjugated tetramers (20 μg/mL). After washes, tetramer-stained cells were enriched using anti-PE antibody-coated immunomagnetic beads on LS columns (Miltenyi Biotech) according to manufactured instructions. After enrichment, cells were stained with an antibody panel that is summarized in Table S2. Counting beads (123count eBeads, eBioscience) were used to normalize results. Cells were acquired on a FACS Fortessa X20 cytometer, a FACS LSR II cytometer, or a FACS Aria III sorter (BD Biosciences) and analyzed with FlowJo software (version 10, Tree Star Inc.). For PE-labeled Tetramer^+^ CD8^+^ T cell detection and phenotyping, CD3^+^ CD8^+^ CD4^−^ T cells were gated on single live dump^−^ (CD14^−^ CD16^−^ CD19^−^) cells. CD45RA, CD45RO, and CCR7 expression was then assessed on PE^+^ APC^−^ CD8^+^ T cells. Statistical analyses were performed using GraphPad Prism 8.0.1. Results were considered non-significant when *p* > 0.05.

### Cell Sorting and T-Cell Lines

A2- or B7-restricted AAV8-specific CD8^+^ T cells were obtained after sorting PE-conjugated Tetramer^+^ CD8^+^ T cells with a FACS Aria III sorter (BD Biosciences). Cells were then expanded *in vitro* under non-specific conditions using rIL-2 (300 UI/mL, Novartis), phytohemagglutinin (PHA, 1 μg/mL, Sigma-Aldrich), irradiated PBMCs, and B lymphoblastoid cells as previously described (14). T cell lines were maintained for 3 weeks without restimulation in RPMI 1640 medium containing 1 mM L-glutamine (Lonza), and supplemented with 10% FCS (Pierce-Hyclone) and 300 UI/mL rIL-2, before analysis. Additional rounds of amplification were performed with the same conditions if needed. Purity and specificity of CD8^+^ T cell lines were checked through staining with AAV8 capsid-specific PE-conjugated tetramers and irrelevant APC-conjugated tetramers.

### Intracellular Cytokine Staining

To assess degranulation activity and cytokine expression of human primary A2- or B7-restricted AAV8-specific CD8^+^ T cell lines, A2^+^ TAP-deficient T2 cells or B7^+^ 7221.221 cells were loaded overnight at 37°C with 10 μg/mL of irrelevant peptides, or with A2- or B7- restricted AAV8 capsid-derived peptides (the same peptides used for TAME). A2- or B7-restricted AAV8-specific CD8^+^ T lymphocytes were then added at an effector / target *ratio* of 1/1. Effector and target cells were then incubated 4 h at 37°C in presence of anti-CD107a and anti-CD107b antibodies (clones H4A3 and H4B4 *resp*., BD Biosciences) and 5 μM of monensin sodium (Sigma-Aldrich). As a positive control, effector cells were stimulated with 10 ng/mL PMA (Phorbol 12-myristate 13-acetate, Sigma) and 250 ng/mL Ionomycin (Sigma). After incubation, cells were stained with eFluor 506-conjugated FVD and eFluor 605-conjugated anti-CD8. Cells were then fixed with PFA 1% (EMS) overnight at 4°C, and permeabilised with permeabilisation buffer (eBioscience). Finally cells were stained with PE-conjugated anti-IFNγ (clone B27, BD Biosciences) and APC-conjugated anti-TNFα antibodies (clone Mab11, BD Biosciences). Analysis was performed using a FACS Fortessa X20 or a FACS LSR II cytometer. For quantification of degranulation activity, CD107 expression was assessed on single live CD8^+^ T lymphocytes. For quantification of cytokine secretion, IFNγ and TNFα secretion was assessed on single live CD8^+^ T lymphocytes. Each cell line was assessed through three independent activation assays.

### Cytotoxic Assay

To assess cytotoxic activity of human primary A2-restricted AAV8-specific CD8^+^ T cell lines, lactacte deshydrogenase (LDH) release following target cell lysis was measured with CytoTox 96^®^ non-radioactive cytotoxicity assay (Promega). 5 × 10^5^ A2^+^ TAP-deficient T2 cells were loaded as described for functional assays. AAV8-specific CD8^+^ T lymphocytes were then added at an effector: target *ratio* of 5:1, 10:1, and 20:1. Effector and target cells were then incubated 4 h at 37°C and LDH release was measured after 4 h of incubation at 37°C. Target cell maximum LDH release was measured by adding medium with 1% Triton X-100. Each condition was made in triplicate. Medium background was subtracted from measurement and lysis percentages were calculated as follow:

% cytotoxicity = (Experimental—Effector spontaneous—Target spontaneous)/(Target Max—Target spontaneous) × 100.

### CD4^+^ or CD8^+^ Depletion

CD4^+^ or CD8^+^ T cells have been depleted from total PBMCs using Stem Cell magnetic-based system (Easy Sep St Easy Sep Stem Cell kits #17852 and #17853) according to manufacturer instructions. Then, efficiency of depletion have been controlled by BD LSR II flow cytometer using anti-CD4 APC-H7 (BD Biosciences, #560251) and CD8 eFluor 605 (eBiosciences, #93-0088-42).

### IFNγ ELISpot Assay

Anti-AAV2 and anti-AAV8 cellular immune responses were evaluated with an IFNγ ELISpot assay using overlapping peptide libraries covering the whole AAV2 or AAV8 capsid sequences (15 per 10 mers, PEPscreen, Sigma) split into three peptide pools each. MultiScreenHTS filter plates, with polyvinyldiene difluoride membrane (PVDF, Millipore) were coated overnight at 4°C with human anti-IFNγ antibody (clone MT126L, Mabtech). After coating, 2 × 10^5^ cells per well were plated and restimulated 48H with AAV2- or AAV8- derived peptide pools at a final concentration of 10 μg/mL. Medium alone served as negative control, while cells stimulated with concanavalin A (Con A, 10 μg/mL, SIGMA) served as a positive control. After incubation with a biotinylated anti-IFNγ antibody (clone 7-B6-1, MabTech) and Streptavidin-ALP (MabTech), enzymatic reaction was revealed using NBT/BCIP (MabTech). Spot number was determined using an ELISpot *iSpot* Spectrum reader (AID, Strassberg, Germany) and analyzed with AID ELISpot reader Software V7.0 (AID, Germany). Responses were considered positive when the number of spot-forming colonies per million cells was >50 and at least threefold higher than the medium alone negative control. For positive responses, statistical analyses were performed using a DFR(2×) test (Distribution Free Resampling).

## Results

### AAV2- and AAV8-Specific CD8^+^ T Cells Are Detectable in Peripheral Blood After *ex vivo* Tetramer Enrichment

Due to their low frequencies, detection of AAV-specific CD8^+^ T lymphocytes often requires prior amplification (or stimulation) of PBMCs or splenocytes (10, 16–18), a process which might induce some bias in the frequency or phenotype of those cells. To circumvent this limitation, we evaluated Tetramer-Associated Magnetic Enrichment (TAME), a flow cytometry-based method that can rapidly and efficiently detect scarce subpopulations of CD8^+^ T cells *ex vivo* (13, 14). We first proved the technical feasibility of this strategy in our hands using HLA-A^*^0201 tetramers loaded with the Melan-A tumor-associated antigen ELAGIGILTV (pELA/A2). While Melan-A-specific CD8^+^ T cells displayed frequencies too low to be detected *ex vivo* through conventional tetramer flow cytometry assay, we were able to systematically detect them after TAME in a cohort of 31 HLA-A2^+^ donors, at frequencies ranging from 35 to 2,600/10^6^ total CD8^+^ T cells (median: 124/10^6^ total CD8^+^ T cells) (Figure S1).

**Figure 1:**
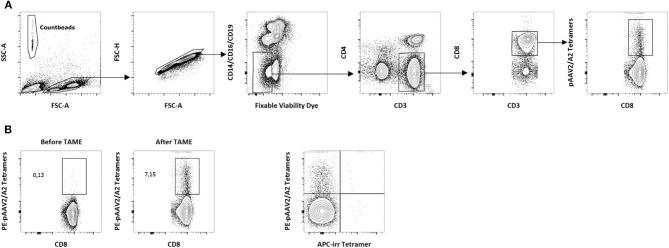
Detection of AAV2-specific CD8^+^ T lymphocytes in PBMCs from A2^+^ donors. **(A)** Gating strategy for the detection of p-MHC tetramers^+^CD8^+^ T cells *ex vivo*. Tetramer enrichments were performed on freshly isolated PBMCs. After exclusion of dead cells, PE-Tetramers^+^ events were assessed within the CD14^−^ CD16^−^ CD19^−^CD3^+^ CD4^−^ CD8^+^ compartment. A representative staining following pAAV2/A2 tetramer enrichment is shown. PE, phycoerythrin. Count beads (123count eBeads, eBioscience) were used to normalize results. **(B)** Representative dot plots obtained before and after tetramer enrichment with pAAV2/A2 complexes. Analyses were performed on CD8^+^ T cells after gating as described above. Percentages of PE-Tetramers^+^ cells among total CD8^+^ T cells obtained before and after tetramer enrichment are indicated. **(C)** Representative dot plot showing the specificity of tetramer staining after tetramer enrichment with pAAV2/A2 complexes. Analyses were performed after gating as described above. An irrelevant APC-conjugated A2-restricted tetramer (APC-irr tetramer) was used as a specificity control. APC, allophycocyanin.

**Figure 2:**
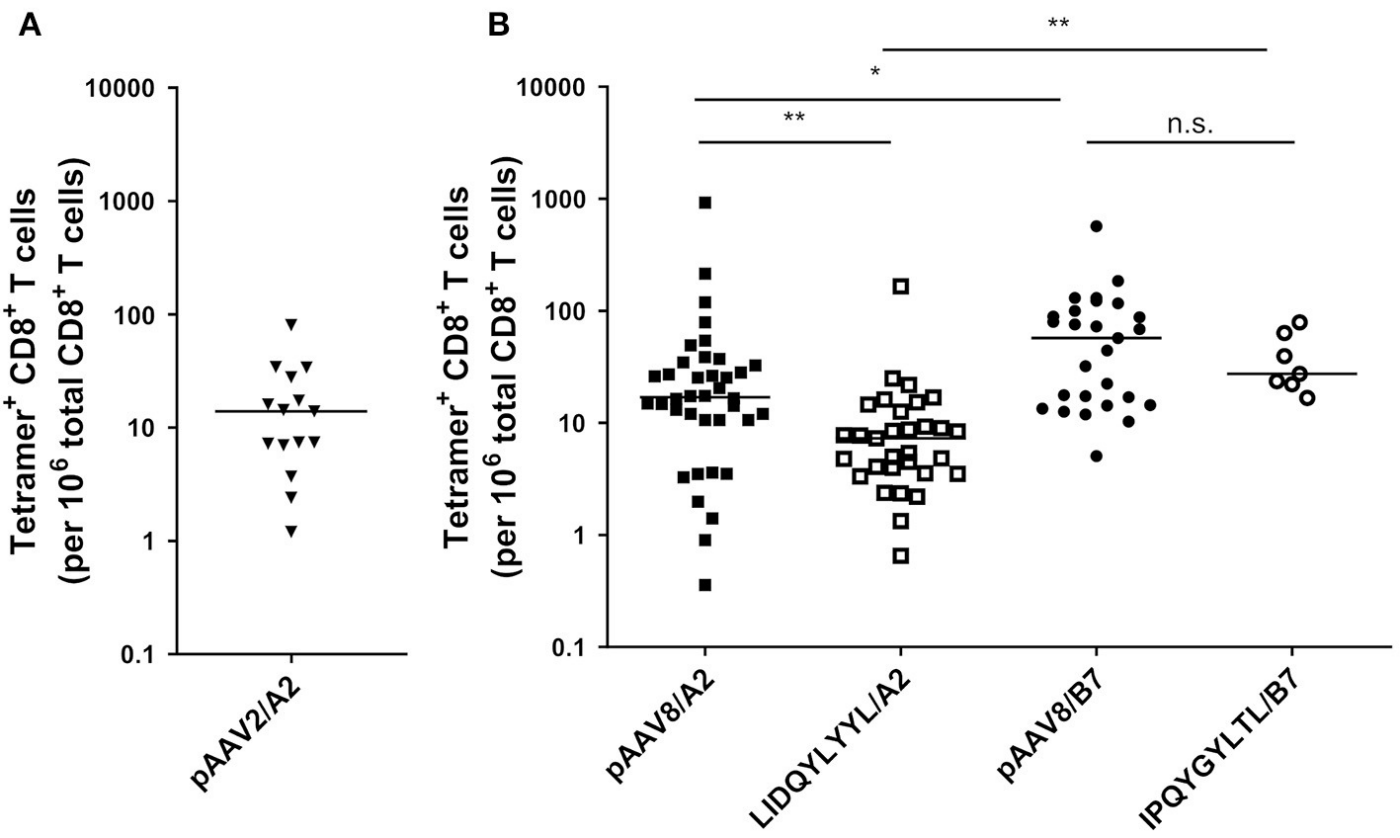
Detection of AAV2- and AAV8-specific CD8^+^ T lymphocytes in PBMCs from A2^+^ and B7^+^ donors. **(A)** *Ex vivo* frequencies of pAAV2/A2 tetramers^+^ CD8^+^ T lymphocytes in A2^+^ donors. **(B)** *Ex vivo* frequencies of pAAV8/A2, LIDQYLYYL/A2 (HLA-A2 restricted immunodominant peptide), pAAV8/B7, and IPQYGYLTL/B7 (HLA-B7 restricted immunodominant peptide) Tetramers^+^ CD8^+^ T lymphocytes in A2^+^ and B7^+^ donors. *Ex vivo* frequencies of AAV2- and AAV8-specific CD8^+^ T cells were calculated by dividing the absolute number of PE-Tetramer^+^ CD8^+^ T cells, detected after tetramer enrichment, by the absolute number of total CD8^+^ T cells. Results are represented as the number of tetramers^+^ CD8^+^ T cells per 10^6^ total CD8^+^ T cells. Horizontal bars refer to median values. Each symbol represents an individual: *n* = 15 for pAAV2/A2, *n* = 38 for pAAV8/A2, *n* = 31 for LIDQYLYYL/A2, *n* = 27 for pAAV8/B7, and *n* = 7 for IPQYGYLTL/B7. Statistical analysis: Kruskall & Wallis; *p < 0.05, **p < 0.01.

As AAV2 was shown to be the most seroprevalent serotype in humans (19), and HLA-A2 the most prevalent allele among the Caucasian population (20), we first investigated the peripheral CD8^+^ T cell repertoire reactivity toward AAV2 capsid-derived peptides restricted to HLA-A^*^0201 allele (referred to as pAAV2/A2, p for pooled peptides) (Table S1). For that purpose, we used soluble phycoerythrin (PE)-labeled pAAV2/A2 tetramers obtained with a previously described UV-mediated peptide exchange technology (15). Thirteen A2-restricted AAV2 capsid peptides were selected using bioinformatical prediction databases tetramer. PBMCs isolated from A2^+^ donors were stained with pAAV2/A2 tetramers (13 tetramers pooled together), enriched with anti-PE para-magnetic microbeads, and analyzed through flow cytometry (Figure 1A for gating strategy; Figure 1B for an example of enrichment). Following TAME, we were able to readily detect CD8^+^ T cells stained with pAAV2/A2 tetramers *ex vivo* in all A2^+^ donors tested (*n* = 15, Figure 2A). Staining with an irrelevant allophycocyanin (APC)-labeled tetramer confirmed the specificity of pAAV2/A2 tetramer staining, as in all assays and for all donor samples, >0.1% of PE^+^ APC^+^ CD8^+^ T cells could be seen (Figure 1C for a representative staining). Absolute numbers of AAV2-specific CD8^+^ T cells were determined to calculate their *ex vivo* frequencies and ranged from 1.2 to 80/10^6^ total CD8^+^ T cells in donors (median: 14/10^6^ total CD8^+^ T cells) (Figure 2A), which is in the range of previously described T cell repertoires directed against some viral or tumoral antigens found in healthy donors (13, 14).

Following this proof of concept, we moved for the rest of the study to the AAV8 serotype as it is today a clinically relevant serotype in muscle- and liver-directed systemic gene transfer (21). Peripheral CD8^+^ T cell repertoire reactivity toward AAV8 capsid-derived antigens restricted by the HLA-A^*^0201 or HLA-B^*^0702 alleles (referred to as A2 or B7, respectively) was studied in healthy donors expressing at least one of those alleles. Using the same strategy described above, we constituted a pool of 9 pAAV8/A2 tetramers and a pool of 12 AAV8/B7 tetramers [notably containing the previously described HLA B7-restricted AAV8 epitope (10) (Table S1)] to identify AAV8-specific CD8^+^ T lymphocytes within the PBMCs of A2^+^ or B7^+^ healthy donors, respectively. Once again, following TAME, we were able to detect CD8^+^ T cells stained with pAAV8/A2 or pAAV8/B7 tetramers in all A2^+^ and B7^+^ donors tested without any amplification or stimulation of PBMCs beforehand. Specificity of the staining was systematically confirmed using irrelevant APC-labeled tetramers. *Ex vivo* frequencies of AAV8-specific CD8^+^ T cells ranged from 0.3 to 920/10^6^ total CD8^+^ T cells in A2^+^ donors (median: 17/10^6^ total CD8^+^ T cells, *n* = 38), and from 5 to 570/10^6^ total CD8^+^ T cells in B7^+^ donors (median: 57/10^6^ total CD8^+^ T cells, *n* = 27) (Figure 2B).

We further assessed the frequency of CD8^+^ T cells specific for the previously described immuno-dominant A2-restricted LIDQYLYYL and B7-restricted IPQYGYLTL peptides (15, 22). These peptides were used to construct PE-labeled tetramers for TAME on PBMCs from A2^+^ or B7^+^ healthy donors, respectively. Similar to our previous results using pooled tetramers with various peptide specificities, we were again able to detect AAV8-specific CD8^+^ T cells stained with single tetramers in all donors. In A2^+^ donors, these cells were detected at frequencies significantly lower than peptides pool (*p* = 0.0089) and comprised between 0.653 and 166/10^6^ total CD8^+^ T cells (median: 7.3/10^6^ total CD8^+^ T cells, *n* = 31). For B7^+^ donors, the frequencies were also slightly lower than peptides pool with no statistical significance: 16–78.8/10^6^ total CD8^+^ T cells in B7^+^ donors (median: 27.4/10^6^ total CD8^+^ T cells, *n* = 7) (Figure 2B). Interestingly, for both peptide pools and immunodominant peptides, AAV8-specific CD8^+^ T cells were found more frequent in B7^+^ donors than A2^+^ samples (Kruskall-Wallis statistical test: *p* = 0.0469 and *p* = 0.0037, respectively).

Overall, we evidenced that all healthy donors tested possessed circulating AAV2 or AAV8-specific CD8^+^ T cells, though at very low frequencies among PBMCs, and that TAME is a suitable and feasible method to allow their *ex vivo* detection, even though it does not provide any functional insight.

### Human Primary AAV8-Specific CD8^+^ T Cells Expanded *in vitro* Secrete Pro-inflammatory Cytokines and Can Specifically Mediate Cytotoxicity

**Figure 3:**
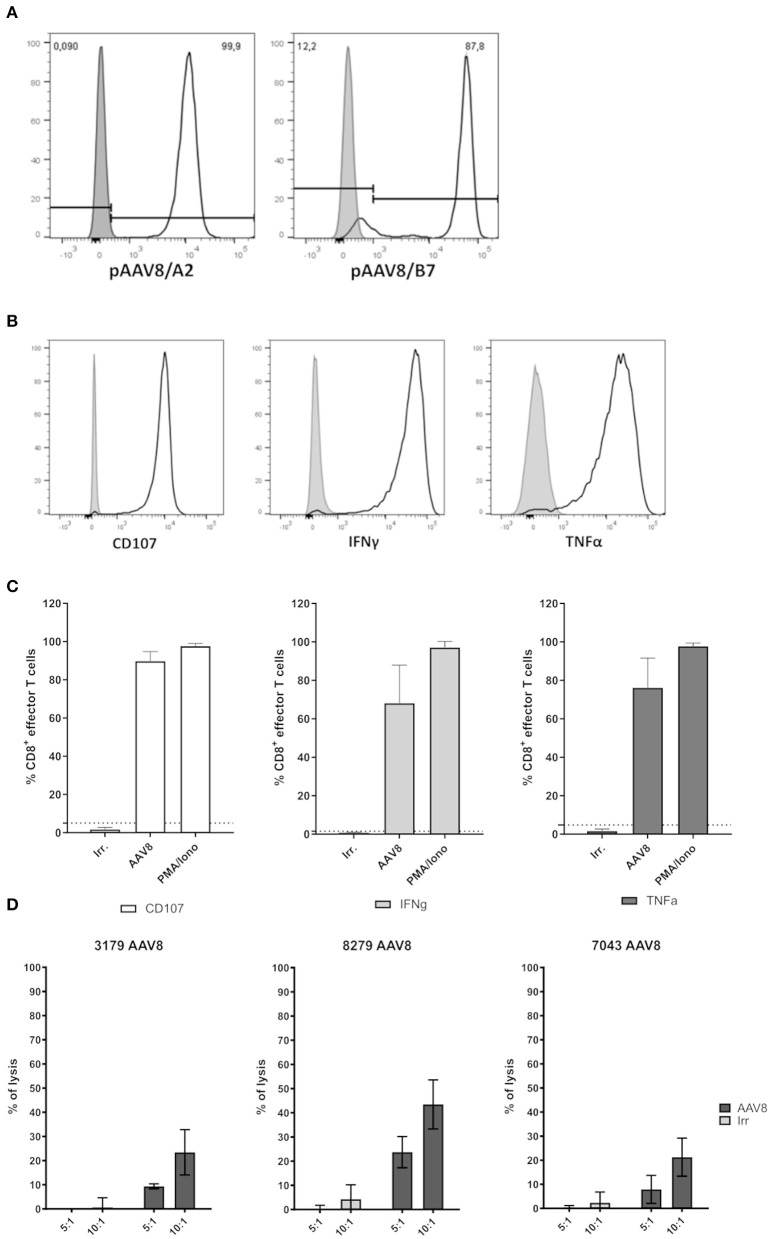
Functional assessment of primary AAV8-specific CD8^+^ T cell lines derived from A2^+^ or B7^+^ donors. **(A)** Representative histograms of pAAV8/A2 or pAAV8/B7 tetramer staining of AAV8-specific CD8^+^ T cell lines generated from A2^+^ or B7^+^ donors. AAV8-specific CD8^+^ T cell lines were stained with relevant (white) or irrelevant (gray) tetramers. The list of all AAV8-specific CD8^+^ T cell lines generated and their purity are indicated in Table 1. **(B)** Representative histograms of AAV8-specific CD8^+^ T cell lines activation. CD107 expression, IFNγ secretion, and TNFα secretion were assessed by flow cytometry on AAV8-specific CD8^+^ T cell lines after 4H of co-culture with target cells loaded with AAV8 peptides (light gray) or irrelevant peptides (dark gray). **(C)** Representative quantification of AAV8-specific CD8^+^ T cell lines activation. Each cell line was assessed through three independent activation assays. Results are represented as the mean percentage of AAV8-specific CD8^+^ T cells expressing CD107, IFNγ, and TNFα in response to target cells loaded with AAV8 peptides (AAV8) or irrelevant peptides (Irr.). Stimulation with PMA/Ionomycin (PMA/Iono) served as positive control. AAV8-specific CD8^+^ T cells were considered positive when the expression of CD107, IFNγ, and TNFα in response to AAV8-loaded target cells was above the threshold of positivity (dotted line) calculated as the percentage mean of positive cells obtained in response to irrelevant peptides (mean + 3 SD). Overall activation profiles are shown in Table 1. **(D)** Representative quantification of AAV8-specific CD8^+^ T cell lines cytoxicity. Each cell line was assessed for cytotoxicity through LDH measurement. Results are represented as the percentage of lysis of target cells loaded with AAV8 peptides (AAV8) or irrelevant peptides (Irr.) by AAV8-specific CD8^+^ T cells for different effector:target ratio (5:1 and 10:1).

In order to investigate the functional properties of AAV8-specific CD8^+^ T cells, tetramer positive CD8^+^ T cells were sorted by flow cytometry after TAME and expanded *in vitro* from PBMCs of A2^+^ or B7^+^ donors. Indeed, low cell numbers were obtained following cell TAME-based enrichment and sorting and did not allow *in vitro* functional assays before amplification. The purity of all tetramer-sorted CD8^+^ T cell lines after expansion was assessed by pAAV8/A2 or pAAV8/B7 tetramer staining and was comprised between 80 and 99%, except for 2 T cell lines with a purity of 71 and 72%, respectively (Figure 3A for representative A2^+^ and B7^+^ tetramer staining on expanded T cell lines; Table 1 for overall summary). AAV8-specific CD8^+^ T cell lines were tested for their functionality in the presence of A2^+^ or B7^+^ target cells loaded with AAV8 peptides vs. irrelevant peptides. The percentage of CD8^+^ effector cells expressing degranulation marker CD107, or secreting IFNγ and TNFα was analyzed by flow cytometry (Figures 3B,C) for representative responses; Table 1 for overall results). Each T cell line was assessed through three independent activation assays. Responses were considered positive when the percentage of CD8^+^ effector cells expressing CD107, IFNγ, and TNFα in response to AAV8-loaded target cells was found above a threshold of positivity determined as the percentage mean of CD8^+^ effector cells positive for those markers in response to target cells loaded with an irrelevant peptide (mean + 3SD) (Figure 3C). Out of the 12 cell lines tested, 11 showed significant upregulation of at least two activation markers upon incubation with AAV8-loaded target cells. Of note, these markers correlated well with each other, as virtually almost all cells upregulating CD107 also expressed IFNγ and TNFα. For three T cell donors, we also investigated the cytotoxic activity of AAV8-specific CD8^+^ T cell lines using LDH measurement (Figure 3D). Each AAV8-specific CD8^+^ T cell line was tested with 2 different effector:target ratio: 5:1 and 10:1. AAV8-specific CD8^+^ T cell lines were able to mediate cytotoxicity specifically in response to AAV8 peptide-loaded target cells and not against irrelevant peptide-loaded target cells. Overall, these results indicate that *in vitro* expanded tetramer positive CD8^+^ T cells can secrete pro-inflammatory cytokines and mediate cytotoxicity against target cells loaded with AAV8 peptides.

**Table 1:** Overview of *in vitro* functional responses obtained from primary AAV8-specific CD8^+^ T cell lines derived from PBMCs of A2^+^ and B7^+^ donors.

			***In vitro*** **functional responses**			
**Cell lines**	**HLA restriction**	**Purity^a^ (%)**	**Upregulation of CD107^b^**	**IFNγ secretion^b^**	**TNFα secretion^b^**	**IFNγ^c^**
4374	HLA-A2	84	Positive	Positive	Positive	Negative
3179		87	Positive	Positive	Positive	Negative
4800		79.4	Positive	Positive	Positive	Negative
9946		98.9	Positive	Negative	Positive	Negative
6062		91.1	Positive	Positive	Positive	Negative
8279		99.3	Positive	Positive	Positive	Negative
3654	HLA-B7	97.4	Positive	Positive	Positive	Negative
2367		84.5	Positive	Positive	Positive	Positive
5576		99.2	Negative	Negative	Negative	Negative
8860		71.1	Positive	Positive	Positive	Negative
7043		82.1	Positive	Positive	Positive	Positive
6714		72.2	Positive	Positive	Positive	Positive
Total			11/12	10/12	11/12	3/12

a *Purity of pAAV8 tetramer-sorted CD8-specific CD8^+^ T cell lines was assessed through staining with relevant tetramers right before functional assessment*.

b *Activation of AAV8-specific CD8^+^ T cell lines was assessed through flow cytometry-based degranulation assay monitoring the upregulation of CD107 expression, IFNγ and TNFα secretion in response to AAV8-loaded target cells. AAV8-specific CD8^+^ T cells were considered positive (gray) when the expression of CD107, IFNγ, and TNFα in response to AAV8-loaded target cells was above the threshold. Positive threshold (dotted line) was calculated using the percentage of positive cells obtained in response to irrelevant peptides (mean + 3 SD)*.

c *The last column represents IFNγ ELISpot responses assessed on whole PBMCs from which the AAV8-specific CD8^+^ T cell lines were derived*.

### AAV8-Specific CD8^+^ T Cells Detected *ex vivo* Contain a T_EMRA_ Effector Memory Subset

**Figure 4:**
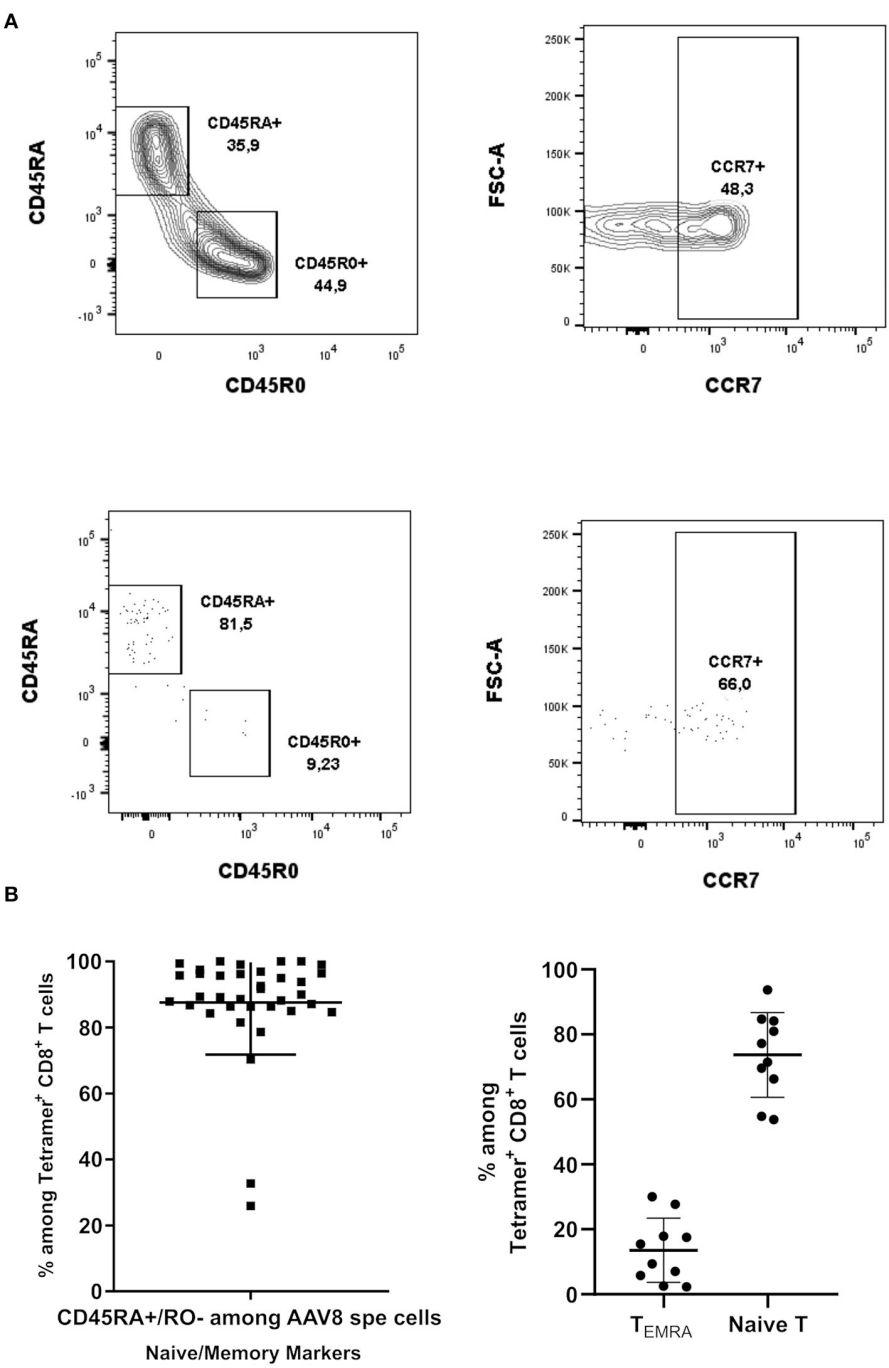
Phenotypical characterization of AAV8-specific CD8^+^ T lymphocytes in PBMCs from A2^+^ and B7^+^ donors. **(A)** Representative dot plots of CD45RA, CD45RO, and CCR7 expression on Tetramer^+^ CD8^+^ T cells. Upper panel: cells were gated on total CD8^+^ T cells. Lower panel: cells were gated on Tetramer^+^ CD8^+^ T cells. **(B)** Overall phenotype of pAAV8 Tetramer^+^ CD8^+^ T cells. Percentages of AAV8-specific CD8^+^ T cells (isolated with AAV8 peptides pool tetramers) displaying a CD45RA^+^/RO^−^. Percentages of AAV8-specific CD8^+^ T cells (isolated with AAV8 immunodominant peptides tetramers) displaying a CCR7^+^ (Naïve T cell) and CCR7^−^ (T_EMRA_) phenotype among CD45RA^+^/RO^−^ T cells are represented. Each symbol represents an individual donor. Horizontal bars refer to median values.

Since CD8^+^ T cell capsid specificity was demonstrated using amplified cells, we further characterized AAV8-specific CD8^+^ T cell phenotype immediately after TAME and without the bias of prior amplification. For that purpose, we assessed the expression of CD45RA, CD45RO, and CCR7 T cell markers by flow cytometry. We first observed that virtually all AAV8-specific CD8^+^ T cells were CD45RA^+^ CD45RO^−^ (Figure 4A, lower panel and Figure 4B). In contrast, total polyclonal CD8^+^ T cells displayed a pool of CD45RO^+^ conventional memory cells, validating the anti-CD45RO antibody staining (Figure 4A, upper panel). To distinguish between the naïve cells and terminally differentiated effector memory (T_EMRA_) phenotype among capsid specific CD45RA^+^ CD45RO^−^ T cells, we next looked at the expression of CCR7 (Figures 4A,B, right panels). CD45RA^+^/RO^−^ CCR7-positive naïve cells ranged from 53.8 to 93.67% (89.65% ± 15.88 SD) of total AAV8-specific CD8^+^ T cells, while CD45RA^+^ CD45RO^−^ CCR7-negative T_EMRA_ cells ranged from 2.27 to 30.07% (12.42 ± 9.8 SD) of total AAV8-specific CD8^+^ T cells. We therefore concluded that while most of the AAV8-specific CD8^+^ T cells detected *ex vivo* had a naïve phenotype, all donors also displayed a subset of antigen-primed T_EMRA_ cells, a phenotype known to be associated with chronic viral infections in humans (23–25).

### *Ex vivo* Frequencies of T_EMRA_ AAV8-Specific CD8^+^ T, but Not Total AAV8-Specific CD8^+^ T Cells, Show a Correlation With Anti-AAV8 IFNγ ELISpot Responses

**Figure 5:**
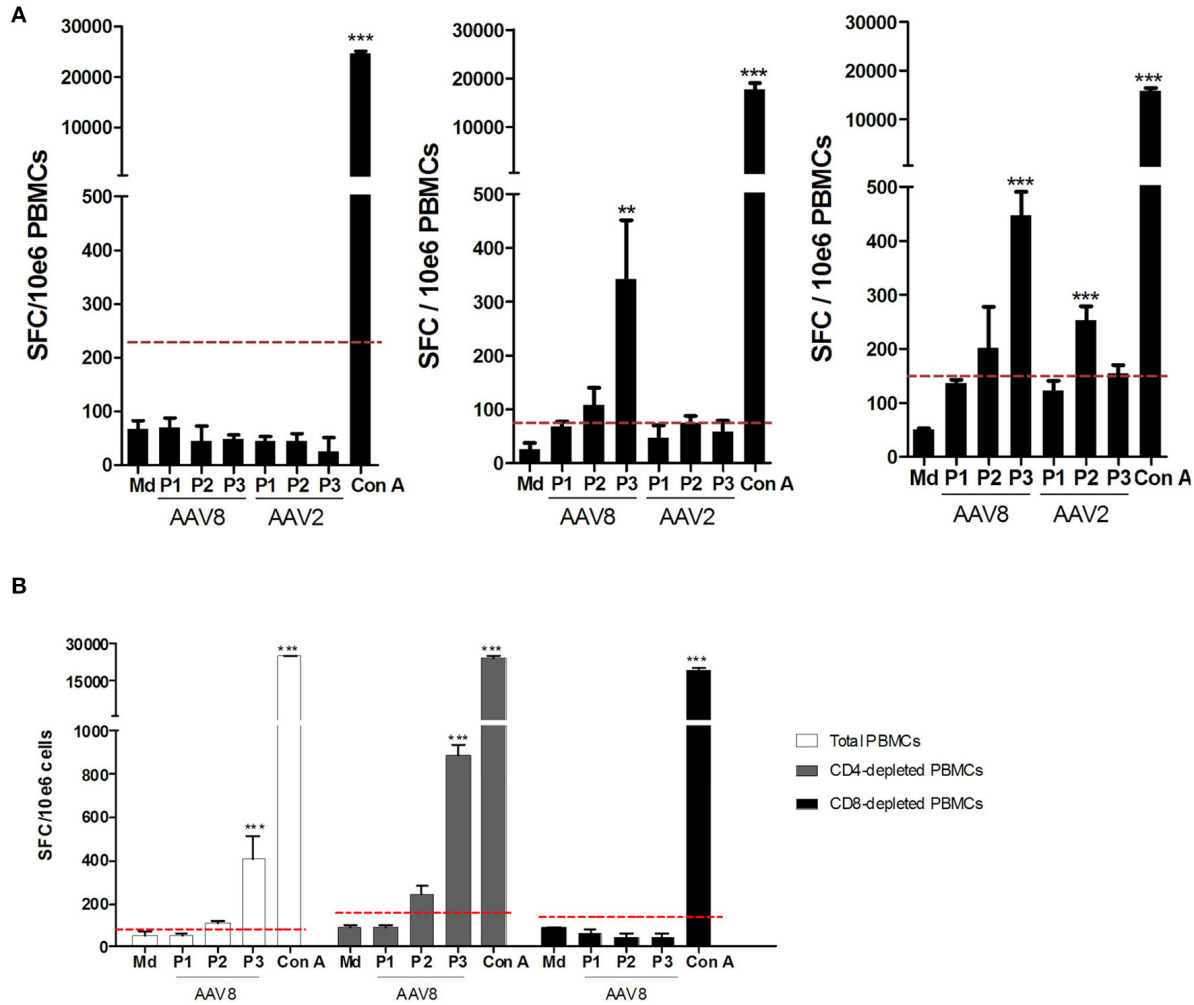
Assessment of anti-AAV2 and anti-AAV8 IFNγ ELISpot responses on PBMCs from A2^+^ and B7^+^ donors. **(A)** Representative anti-AAV8 and anti-AAV2 IFNγ ELISpot responses. Anti-AAV2 and anti-AAV8 IFNγ ELISpot responses were assessed after 48H-stimulation of whole PBMCs from A2^+^ or B7^+^ donors with three pools of overlapping peptides (P1, P2, P3) covering the whole AAV2 or AAV8 capsid sequences. As negative control, PBMCs were cultured in medium alone (Md). Concanavalin A (Con A)-stimulated PBMCs served as positive control. Left: representative donor not responding to AAV2 nor AAV8. Middle: representative donor responding to AAV8 only. Right: representative donor responding to AAV2 and AAV8. Results are represented as the number of Spot Forming Cells (SFC) per million PBMCs. Positive threshold (dotted line) was calculated as three times the value obtained with medium alone or at least 50 SFC/10^6^ cells. Statistical significance was determined using the DFR(2x) statistical test. **p < 0.01. ***p < 0.001. **(B)** Representative anti-AAV8 and anti-AAV2 IFNγ responses detected after CD4^+^ or CD8^+^ T cell depletion of PBMCs. CD4^+^ or CD8^+^ T cells were depleted from PBMCs of A2^+^ or B7^+^ donors through magnetic sorting, prior to IFNγ ELISpot assays which were performed as indicated above. Purity of CD4- or CD8-depleted fractions was assessed through flow cytometry (data not shown).

We next wanted to parallel our results with the IFNγ ELISpot assay which is the conventional method used in clinical trials to monitor anti-AAV capsid cellular responses (26). Anti-AAV2 and anti-AAV8 IFNγ ELISpot assays were performed on all donors (A2^+^ and B7^+^) assessed through TAME using total PBMC as classically used for patient immunomonitoring. PBMCs from those donors were stimulated with 3 pools of overlapping peptides covering the whole AAV2 or AAV8 capsids (Figure 5A for a representative assay). Results from the whole cohort are summarized in Table 2. Overall, 24.7% of the donors responded to AAV8 and 6.2% responded to AAV2. Unexpectedly, all donors responding against AAV2 responded also against AAV8. The majority of positive responses against AAV8 capsid were observed with peptide pools 2 and 3 (Table 2). Pool 2 contains the majority of peptides selected for AAV2-A2 tetramers (10/13) and for AAV8-A2 tetramers (5/9). Immunodominant peptides LIDQYLYYL and IPQYGYLTL are also in pool 2. Peptides selected for AAV8-B7 tetramers are contained in majority in pool 3 (5/12) and pool 2 (4/12) (Table S1). A more detailed analysis of our cohort revealed that B7^+^ donors seem to display a higher rate of positive anti-AAV8 IFNγ ELISpot responses compared to A2^+^ donors (40.0 vs. 28.6% responders, respectively), an observation that parallels the results obtained after TAME where higher *ex vivo* frequencies of AAV8-specific CD8^+^ T cells were observed in B7^+^ donors (Table 2 and Figure 2B). It is nevertheless important to note that TAME and ELISpot methods have different read-outs (tetramer staining on one hand, IFNγ secretion on the other hand using total PBMC).

**Table 2:** Overview of anti-AAV8 and anti-AAV2 IFNγ responses assessed on PBMCs from A2^+^ and B7^+^ donors.

**ANTI-AAV8 AND ANTI-AAV2 IFNγ** **RESPONSES**						
		**Positivity**		**Cross-reactivity**		
**HLA alleles**	**Donors tested**	**AAV2 positive**	**AAV8 positive**	**AAV2 only**	**AAV8 only**	**AAV2 and AAV8**
A2^+^/B7^−^	43	1 (2.3%)	5 (11.6%)	0	4 (9.3%)	1 (2.3%)
A2^−^/B7^+^	15	2 (13.3 %)	8 (53.3%)	0	6 (40.0%)	2 (13.3%)
A2^+^/B7^+^	7	1 (14.3 %)	3 (42.8%)	0	2 (28.6%)	1 (14.3%)
Total	65	4 (6.1%)	16 (24.6%)	0	12 (18.5%)	4 (6.2%)
**ANTI-AAV8 IFNγ** **RESPONSES**						
**HLA alleles**	**Donors responding against AAV8**			**Pool 1**	**Pool 2**	**Pool 3**
A2^+^/B7^−^	5			2 (40%)	4 (80%)	2 (40%)
A2^−^/B7^+^	8			0 (0%)	3 (37,5%)	8 (100%)
A2^+^/B7^+^	3			1 (33.3%)	1 (33.3%)	3 (100%)
Total	16			3 (18.7%)	8 (50%)	12 (75%)
**ANTI-AAV2 IFNγ** **RESPONSES**						
**HLA alleles**	**Donors responding against AAV2**			**Pool 1**	**Pool 2**	**Pool 3**
A2^+^/B7^−^	1			0 (0%)	1 (100%)	0 (0%)
A2^−^/B7^+^	2			0 (0%)	2 (100%)	1 (50%)
A2^+^/B7^+^	1			0 (0%)	1 (100%)	0 (0%)
Total	4			0 (0%)	4 (100%)	1 (25%)

To further correlate the two assays, we first refined the analysis of anti-AAV cellular responses obtained through ELISpot assays by investigating which circulating lymphocyte sub-population (CD4^+^ or CD8^+^) was responsible for IFNγ secretion. Consequently, PBMCs from anti-AAV IFNγ ELISpot responders were tested a second time, but were subjected to CD4^+^ or CD8^+^ T cell depletion beforehand (Figure 5B for a representative donor). The efficiency of CD4 and CD8 depletions was confirmed through flow cytometry (purity from 94.27 to 99.71% for CD8-depleted fraction and 90.52 to 99.64% for CD4-depleted fraction). In all donors thus tested, CD4-depleted PBMCs responded in the same way as total PBMCs (with a higher sensibility even), while in CD8-depleted PBMCs the response was completely abrogated. These results suggest that AAV-directed IFNγ ELISpot responses were primarily mediated by CD8^+^ T cells.

**Figure 6:**
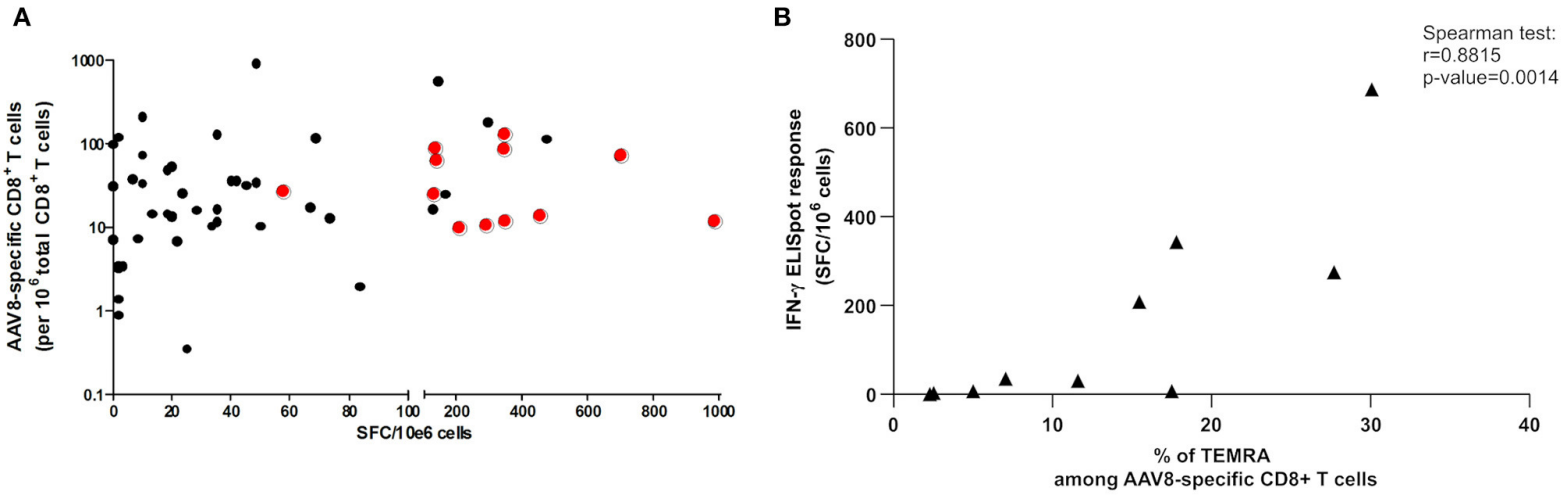
Correlation studies between TAME and IFNγ ELISpot results. **(A)** Correlation study between *ex vivo* frequencies of AAV8-specific CD8^+^ T cells and anti-AAV8 IFNγ ELISpot responses. *Ex vivo* frequencies of AAV8-specific CD8^+^ T cells, determined after tetramer enrichment, were plotted against SFC/10e6 cells values obtained through anti-AAV8 IFNγ ELISpot assays. Red dots represent the positive responses (above threshold) in ELISpot assays. Spearman correlation test revealed no correlation between both parameters. **(B)** Correlation study between percentage of T_EMRA_ cells among AAV8-specific CD8^+^ T cells and anti-AAV8 IFNγ ELISpot responses. Percentage of T_EMRA_ cells among AAV8-specific CD8^+^ T cells determined after tetramer enrichment, were plotted against SFC/10e6 cells values obtained through anti-AAV8 IFNγ ELISpot assays. Spearman correlation test revealed a correlation between both parameters (*p* = 0.0014).

We then tested the possibility that anti-AAV responses detectable by IFNγ ELISpot assay stemmed from donors displaying the highest *ex vivo* frequencies determined after TAME. We therefore plotted tetramer^+^ AAV8-specific CD8^+^ T cell frequencies against anti-AAV8 IFNγ ELISpot responses (Figure 6A). We focused on AAV8 responses for which the total number of donors tested with tetramer pools reached 65 individuals. No obvious correlation could be found between the parameters assessed, and particularly, highest frequencies did not reflect in ELISpot responses above the threshold of positivity (represented by the red dots in Figure 6A).

Finally, we tested the correlation between the proportion of CD45RA^+^/RO^−^ CCR7^−^ T_EMRA_ memory cells after TAME selection and IFNγ ELISpot responses for AAV8 serotype. We observed a positive correlation between the two parameters validated through a Spearman correlation test (Figure 6B). This finding suggests that T_EMRA_ specific cells could be among the functional IFNγ-secreting T cell population after capsid AAV8 stimulation.

## Discussion

Up until now, phenotypical characterization of AAV-specific CD8^+^ T cells has almost exclusively relied on expansion or stimulation of PBMCs or splenocytes prior to phenotypical or functional assessment (10, 17, 18). Here, to circumvent biases inherent to *in vitro* cell amplification or cell stimulation (26), we used the highly sensitive pMHC-based tetramer enrichment method (TAME) to assess *ex vivo* frequencies and phenotypes of AAV2- and AAV8-specific CD8^+^ T cells using PBMCs of healthy A2^+^ and B7^+^ donors. We detected AAV2- and AAV8-specific CD8^+^ T cells in all donors tested (Figures 2A,B). Considering that anti-AAV seroprevalence studies have shown that at most 2/3 of human cohorts seem to have encountered WT AAV during their life (20), it is likely that we have detected naïve T cells that are not AAV-capsid antigen experienced as reported for other viral epitopes (13). Moreover, we cannot exclude that in some donors the tetramer-stained cells detected stem from potential HLA-cross reactive CD8 T cells as already shown for tumoral antigens using the same TAME method (27, 28). Indeed, a large number of cross reactive specific T cells recognizing epitopes within different proteins of the same pathogen, proteins from unrelated pathogens or even self-proteins have now been documented in both human or murine settings (22, 29–35). In our study, the detection of capsid tetramer-positive CD8 T cells in all donors might be explained by the fact that pools of tetramers relating to different epitope specificities were used for TAME, though when tetramer enrichment was performed using only the A2 or B7-restricted immunodominant epitope, both subsets were still detectable. The frequencies at which we detected AAV capsid-specific CD8^+^ T cells are coherent with other studies using TAME to assess pre-immune CD8^+^ T cell repertoires or virus-specific CD8^+^ T cells in the frame of chronic viral infections, i.e., situations where the frequencies of CD8^+^ T cells are too low to be evaluated using conventional flow cytometry (36–38). It is also likely that the frequencies of AAV-specific CD8^+^ T cells we calculated are somewhat underestimated, the main reasons being: (1) the potential loss of cells during the experimental procedure (14); (2) the AAV2- and AAV8-derived peptides selected to construct the tetramers used for TAME (Table S1). Indeed, out of the many epitopes derived from AAV2 or AAV8 capsids, only the first 30 with the highest bioinformatics prediction scores were retained for UV-mediated peptide exchange screening. In the end, only 13 peptides served to construct pAAV2/A2 tetramers, nine peptides for pAAV8/A2's, and 12 peptides for pAAV8/B7's (Table S1). Of note, among the B7-restricted epitopes, our selection contained the immunodominant epitope identified by Mingozzi et al. (10), a proof of the soundness of our bioinformatical epitope selection strategy. Therefore, we are far from covering the whole CD8^+^ T cell repertoire potentially associated to capsid antigenicity. Regarding serotype-specific response, the reactivity against the non-human primate serotype AAV8 in our cohort could also reflect epitope cross-reactivity as AAV8 capsid was shown highly cross-reactive with other AAV serotypes (10, 39). This is why we have not included A2-restricted AAV2 peptides in AAV8 peptide pools which could further underestimate specific CD8 T cell frequencies (Table S1).

During rAAV-based gene therapy clinical trials, destruction of transduced cells was shown to result from reactivation of capsid-specific cytotoxic CD8^+^ lymphocytes which had previously been established following natural infections with wild-type AAV (40). Consistent with this working hypothesis was the observation among different human populations that capsid-specific CD8^+^ T cells could exhibit a resting central memory phenotype (10), a central memory phenotype (17), or an effector memory phenotype (18). Nevertheless, up to now, phenotyping of capsid-specific CD8^+^ T cells has generally been performed after *in vitro* stimulation of PBMCs. Here, we addressed *ex vivo* phenotype of capsid-specific CD8^+^ T cells without prior stimulation and/or expansion, and we evidenced two subpopulations: naïve CD8^+^ T cells (CD45RA^+^/RO^−^ CCR7^+^) and T_EMRA_ effector memory CD8^+^ T cells (CD45RA^+^/RO^−^ CCR7^−^) (Figure 4). The discrepancy we observed with previous studies (where CD45RO^+^ cells were detected) could be explained by the difference in human populations that were analyzed, but could also stem from the fact that we assessed phenotype *ex vivo*, while other studies did so after *in vitro* stimulation. Indeed, in our hands, when we expanded primary AAV8-specific CD8^+^ T cell lines from PBMCs, we observed a change in phenotype, with virtually all cell lines switching from CD45RA^+^/RO^−^ to CD45RA^−^/RO^+^ phenotype (data not shown). The detection of capsid-specific T_EMRA_ effector memory cells in the AAV context could help understanding the natural history of AAV infection in humans. T_EMRA_ effector memory CD8^+^ T cells represent the most differentiated memory T cell subset. They exhibit low proliferative capacities, but they express high levels of IFNγ and cytotoxic molecules, such as perforin and Fas ligand, upon TCR activation (26, 41). It is generally admitted that the presence of T_EMRA_ effector memory CD8^+^ T cells results from chronic exposure of CD8^+^ T cells to their antigens, as can be the case in particular clinical contexts such as graft-vs. -host disease (41), cardiac transplantation (42), chronic viral infections with CMV, HIV, or HCV (23–25) or in AAV infection (35). While the chronicity of WT AAV infection has never been clearly established, a putative evidence of its occurrence is the observation that a significant portion of individuals, aged >60 years old, who harbor anti-AAV1 IgG antibodies also exhibit anti-AAV1 IgM antibodies, an immunoglobulin subtype classically encountered during the course of a running infection (43, 44). Our own results seem to indicate that some AAV8-specific CD8^+^ T cells might be chronically exposed to their antigens, however whether this occurs through WT AAV reinfection cycles or through helper-mediated re-expression of capsid antigens from latent AAV genomes is still up to debate. The detection of whole infectious AAV particles in the bloodstream or in tissues would constitute the most accurate proof of ongoing AAV infections. However, this approach is hardly feasible considering the sensitivity of the detection techniques, and instead it is the presence of infectious AAV DNA in cells that is assessed (45–51).

The presence of circulating naïve self-specific and virus-specific CD8^+^ T cells in contexts where these cells should have exhibited antigen experience has already been evidenced (27, 36). It is now generally known that the distribution of virus-specific CD8^+^ subsets (naïve, central memory, effector memory, T_EMRA_ effector memory) is closely related to the course of viral natural infection and virus wild-type's life cycle, particularly in cases where viruses are able to establish chronic infections or immune tolerance/exhaustion. A history of chronic and repeated infections could also shift CD8 T cell compartment toward the newly described virtual memory subpopulation (T_VM_) in humans (52, 53). T_VM_ is an emerging T cell memory concept where T cells are phenotypically naïve-like (CD45RA+) whereas they harbor memory-like and antigen-specific features allowing an immune response. They were described as negative for CCR7 as T_EMRA_ cells and could be confounded with this T cell memory population. A potential contribution of T_VM_ cells cannot be excluded in our context but still remains to be explored. Additional markers should be used to differentiate true naïve and memory or memory-like T cells. Moreover, because very few things are known about the natural life cycle of WT AAV throughout lifetime (that might in addition differ among serotypes), it is still very difficult to infer immunological status to state of infection in the context of AAV-specific immunity.

To assess the specificity and functionality of AAV8 capsid-reactive CD8^+^ T cells, we sorted cells after TAME to expand primary AAV8-specific CD8^+^ T cell lines *in vitro*. Out of the 11 cell lines that we generated, 10 were responsive, i.e., were able to upregulate CD107, IFNγ and TNFα upon incubation with AAV8-loaded target cells (Figures 3B,C and Table 1). Three cell lines were also tested for cytotoxicity and exhibit a cytotoxic activity against AAV8 peptides loaded cells (Figure 3B). These results were in line with the results obtained by Kuranda et al. who observed secretion of granzyme B by T_EMRA_ and T_EM_ cells in response to stimulation with AAV2 peptides (35).

We noted higher, but not significant, *ex vivo* frequencies of AAV8-specific CD8^+^ T cells in B7^+^ donors compared to A2^+^ individuals, an observation paralleled for anti-AAV8 IFNγ ELISpot responses where responders were more frequent in B7^+^ than in A2^+^ subjects (Figure 2A and Table 2). Interestingly, the first report of AAV2 capsid-specific immunity in hemophilia B clinical trial happened to have been described in a B7^+^ patient (10). Whether B7^+^ individuals are significantly more likely to develop anti-AAV cellular responses, and as such should be more carefully monitored during clinical trials, is a question that remains to be investigated.

While TAME permitted the detection of AAV2- or AAV8-specific CD8^+^ T cells in A2^+^ or B7^+^ donors PBMCs, only 24.6% of donors on the whole were positive when evaluated by anti-AAV2 and anti-AAV8 IFNγ ELISpot assays (Figure 2 and Table 2). Surprisingly, when assessed through IFNγ ELISpot assays, cellular anti-AAV8 responses appeared to be more frequent than anti-AAV2's in our cohort (Table 2). This is unexpected because: (1) the wild-type AAV8 serotype was first isolated from non-human primate samples, and is thought to circulate at a lower rate among the human population (48); (2) according to the literature, AAV2 has always shown a higher seroprevalence than AAV8 in humans (20). Concerning AAV-directed cellular responses, all donors responding to AAV2 peptide pool stimulation also responded to AAV8, which again likely reflects a cross-reactive response, though not complete, between both serotypes, as discussed above, and has been already reported in other studies (39). In our study, anti-AAV8 IgG antibodies were detected in 51.7% of donors (median titer: 1:2,560) and neutralizing factors were detected in 50% of donors (median titer: 1:1,000) (Table S3). Overall, in our cohort, anti-AAV8 humoral responses surprisingly seemed to be more prevalent than in the literature where seroprevalence was generally reported between 20 and 40% (19, 20, 54, 55). A positive correlation was found between anti-AAV2 and anti-AAV8 IgG antibody titers (data not shown) confirming again the cross-reactivity between the two serotypes. When investigating the correlations between anti-AAV8 cellular and humoral responses, we found no correlation between antibody titers and *ex vivo* frequencies of AAV8-specific CD8^+^ T cells assessed by TAME (Figure S2A) or anti-AAV8 IFNγ ELISpot responses (Figure S2B). In addition, frequencies of AAV8-specific T cells assessed by TAME were found quite similar between AAV8 IgG positive and negative donors (Figure S2C). These observations parallel the results already reported for AAV1 (18) and AAV2 (10). Regarding the discrepancy between anti-capsid humoral vs. cellular immune responses, a recent study highlighted two new distinct profiles (56). Indeed, in seronegative individuals, they have shown transient activation of a subset of IFNγ-secreting CD16^bright^CD56^dim^ NK cells known to rapidly respond to infection without prior sensitization. In contrast, seropositive “AAV-experienced” individuals presented capsid-specific memory CD8^+^ T cells that secrete TNFα upon capsid peptide stimulation.

*Ex vivo* frequencies of total AAV-specific CD8^+^ T cells were not predictive of the functionality of AAV-specific CD8^+^ T cells, at least in terms of IFNγ secretion, as we could not evidence any correlation between TAME results and anti-AAV IFNγ ELISpot responses. Appraisal of other parameters [TNFα as reported by Kuranda et al. (56), Granzyme B, Perforin, IL-10…] might have yielded to different conclusions. In addition, we noticed that depletion of CD4^+^ T cells prior to ELISpot assay noticeably increased the intensity of responses (which is expected as when CD4^+^ T cells are removed, CD8^+^ T cells are proportionally more frequent in samples), without increasing the background signal (Figure 5B). As such, CD4^+^ T cell depletion is a quick and an efficient method that might be used to enhance ELISpot detection sensitivity before or during rAAV-mediated clinical trials immunomonitoring.

Interestingly, we evidenced a correlation (*p* = 0.0042) between the proportion of T_EMRA_ effector memory cells within AAV-specific CD8^+^ T cells detected through TAME method and IFNγ positive ELISpot responses using total PBMCs. As we have shown that IFNγ secretion is mediated by CD8 T cells in all positive donors tested using CD4-depleted PBMC (*n* = 16, Figure 5), this correlation is likely evidenced between comparable T cell populations. Because TNFα was recently shown to play a role in preexisting T cells immunity (56), it could be interesting to determine whether TNFα secretion by capsid specific CD8^+^ T cells is also related to a potential TEMRA phenotype. Whether anti-capsid effector responses reported in gene therapy trials is due to the reactivation of T_EMRA_ preexisting cells remains to be investigated. For instance, higher proportion of CD8^+^ T_EMRA_ cells in early phase of HIV infection were shown to be associated to superior antiviral activity and lower viral load (57). It remains important to better characterize AAV-specific T_EMRA_ CD8^+^ T cells to determine their functionality and how they are potentially recalled after administration of rAAV. Indeed, because AAV could result in a viral chronic infection setting as CMV or EBV with changes in immunological memory (24), T_EMRA_ CD8^+^ cells could have an exhausted/non-effector functional phenotype with PD1 regulation in some individuals or infection settings. This could explain why all patients displaying AAV positive IFNγ ELISpot assay did not show transaminitis and subsequent loss of transgene expression in hemophilia gene therapy trials (58).

In conclusion, overall, our study evidenced the difficulty to predict the impact of AAV capsid-specific cellular immunity on the safety and efficiency of rAAV-based gene transfer as it is not only a matter of detection sensitivity, but also a matter of functionality. Both qualitative and quantitative aspects of the CD8^+^ T-cell response need to be considered. Therefore, the development of assays combining both improved detection sensitivity and multiparametric polyfunctional assessment should prove to be the way to efficient clinical pre-screening and monitoring of patients. Emerging data from current and future rAAV-based clinical trials would help understanding T cell memory biology in the context of AAV immunity and its impact on the outcome of gene transfer following the delivery of recombinant AAV vectors. Ultimately, as others before us have underlined, setting up an animal model recapitulating observations done in humans is still an ongoing quest for the field.

## Data Availability Statement

The datasets generated and/or analyzed during the study are available from the corresponding author on reasonable request.

## Ethics Statement

Ethical approval was not required for this study on human participants because blood human samples from healthy donors (cytapheresis) were obtained after written informed consent and under an ethical agreement with the local Etablissement Français du Sang (EFS Nantes, Pays de la Loire, agreement N° PLER NTS 2016-25). The samples are only used for a research purpose.

## Author Contributions

CV, XS, PM, and OA conceived the scientific strategy. CV, RX, LH, XS, MD, and OA a conceived and designed the experiments. CV, RX, MD, NJ, MG, CC, and JL performed the experiments. CV, RX, MD, NJ, MG, CC, and OA analyzed the data. CV, RX, MG, OA, XS, and LH contributed to the writing of the manuscript.

### Conflict of Interest

The authors declare that the research was conducted in the absence of any commercial or financial relationships that could be construed as a potential conflict of interest.

## References

[1] Maguire AM High KA Auricchio A Wright JF Pierce EA Testa F. Age-dependent effects of RPE65 gene therapy for Leber's congenital amaurosis: a phase 1 dose-escalation trial. Lancet. (2009) 374:1597–605. 10.1016/S0140-6736(09)61836-5 19854499 PMC4492302

[2] Bainbridge JWB Mehat MS Sundaram V Robbie SJ Barker SE Ripamonti C. Long-term effect of gene therapy on Leber's congenital amaurosis. N Engl J Med. (2015) 372:1887–97. 10.1056/NEJMoa1414221 25938638 PMC4497809

[3] Nathwani AC Reiss UM Tuddenham EGD Rosales C Chowdary P McIntosh J. Long-term safety and efficacy of factor IX gene therapy in hemophilia B. N Engl J Med. (2014) 371:1994–2004. 10.1056/NEJMoa1407309 25409372 PMC4278802

[4] Mueller C Chulay JD Trapnell BC Humphries M Carey B Sandhaus RA. Human Treg responses allow sustained recombinant adeno-associated virus–mediated transgene expression. J Clin Invest. (2013) 123:5310–8. 10.1172/JCI70314 24231351 PMC3859421

[5] Ferreira V Twisk J Kwikkers K Aronica E Brisson D Methot J. Immune responses to intramuscular administration of alipogene tiparvovec (AAV1-LPL(S447X)) in a phase II clinical trial of lipoprotein lipase deficiency gene therapy. Hum Gene Ther. (2014) 25:180–8. 10.1089/hum.2013.169 24299335 PMC3955976

[6] Melchiorri D Pani L Gasparini P Cossu G Ancans J Borg JJ. Regulatory evaluation of Glybera in Europe - two committees, one mission. Nat Rev Drug Discov. (2013) 12:719. 10.1038/nrd3835-c1 23954897

[7] Russell S Bennett J Wellman JA Chung DC Yu Z-F Tillman A. Efficacy and safety of voretigene neparvovec (AAV2-hRPE65v2) in patients with RPE65-mediated inherited retinal dystrophy: a randomised, controlled, open-label, phase 3 trial. Lancet. (2017) 390:849–60. 10.1016/S0140-6736(17)31868-8 28712537 PMC5726391

[8] Hoy SM. Onasemnogene abeparvovec: first global approval. Drugs. (2019) 79:1255–62. 10.1007/s40265-019-01162-5 31270752

[9] Manno CS Glenn F Arruda VR Pierce GF Glader B Ragni M Successful transduction of liver in hemophilia by AAV-Factor, I. X, and limitations imposed by the host immune response. Nat Med. (2006) 12:342–7. 10.1038/nm1358 16474400

[10] Mingozzi F Maus MV Hui DJ Sabatino DE Murphy SL Rasko JEJ. CD8+ T-cell responses to adeno-associated virus capsid in humans. Nat Med. (2007) 13:419–22. 10.1038/nm1549 17369837

[11] Nathwani AC Tuddenham EGD Rangarajan S Rosales C McIntosh J Linch DC. Adenovirus-associated virus vector–mediated gene transfer in hemophilia B. N Engl J Med. (2011) 365:2357–65. 10.1056/NEJMoa1108046 22149959 PMC3265081

[12] Pien GC Basner-Tschakarjan E Hui DJ Mentlik AN Finn JD Hasbrouck NC. Capsid antigen presentation flags human hepatocytes for destruction after transduction by adeno-associated viral vectors. J Clin Invest. (2009) 119:1688–95. 10.1172/JCI36891 19436115 PMC2689109

[13] Alanio C Lemaitre F Law HKW Hasan M Albert ML. Enumeration of human antigen-specific naive CD8+ T cells reveals conserved precursor frequencies. Blood. (2010) 115:3718–25. 10.1182/blood-2009-10-251124 20200354

[14] Legoux F Debeaupuis E Echasserieau K De La Salle H Saulquin X Bonneville M. Impact of TCR reactivity and HLA phenotype on naive CD8 T cell frequency in humans. J Immunol. (2010) 184:6731–8. 10.4049/jimmunol.1000295 20483723

[15] Rodenko B Toebes M Hadrup SR van Esch WJE Molenaar AM Schumacher TNM. Generation of peptide-MHC class I complexes through UV-mediated ligand exchange. Nat Protoc. (2006) 1:1120–32. 10.1038/nprot.2006.121 17406393

[16] Chirmule N Propert K Magosin S Qian Y Qian R Wilson J. Immune responses to adenovirus and adeno-associated virus in humans. Gene Ther. (1999) 6:1574–83. 10.1038/sj.gt.3300994 10490767

[17] Li H Lasaro MO Jia B Lin SW Haut LH High KA. Capsid-specific T-cell responses to natural infections with adeno-associated viruses in humans differ from those of nonhuman primates. Mol Ther. (2011) 19:2021–30. 10.1038/mt.2011.81 21587208 PMC3222540

[18] Veron P Leborgne C Monteilhet V Boutin S Martin S Moullier P. Humoral and cellular capsid-specific immune responses to adeno-associated virus type 1 in randomized healthy donors. J Immunol. (2012) 188:6418–24. 10.4049/jimmunol.1200620 22593612

[19] Calcedo R Vandenberghe LH Gao G Lin J Wilson JM. Worldwide epidemiology of neutralizing antibodies to adeno-associated viruses. J Infect Dis. (2009) 199:381–90. 10.1086/595830 19133809 PMC10826927

[20] Louis Jeune V Joergensen JA Hajjar RJ Weber T. Pre-existing anti-adeno-associated virus antibodies as a challenge in AAV gene therapy. Hum Gene Ther Methods. (2013) 24:59–67. 10.1089/hgtb.2012.243 23442094 PMC3732124

[21] Balakrishnan B Jayandharan G. Basic biology of adeno-associated virus (AAV) vectors used in gene therapy. Current Gene Therapy. (2014) 14:86–100. 10.2174/1566523214666140302193709 24588706

[22] Evavold BD Sloan-Lancaster J Wilson KJ Rothbard JB Allen PM. Specific T cell recognition of minimally homologous peptides: evidence for multiple endogenous ligands. Immunity. (1995) 2:655–63. 10.1016/1074-7613(95)90010-1 7540944

[23] Eberhard JM Ahmad F Hong HS Bhatnagar N Keudel P Schulze Zur Wiesch J. Partial recovery of senescence and differentiation disturbances in CD8+ T cell effector-memory cells in HIV-1 infection after initiation of anti-retroviral treatment. Clin Exp Immunol. (2016) 186:227–38. 10.1111/cei.12837 27377704 PMC5054564

[24] Shen T Zheng J Xu C Liu J Zhang W Lu F. Pd-1 expression on peripheral Cd8+ tem/temra subsets closely correlated with Hcv viral load in chronic hepatitis C patients. Virol J. (2010) 7:310. 10.1186/1743-422X-7-310 21070674 PMC2989324

[25] Weltevrede M Eilers R de Melker HE van Baarle D. Cytomegalovirus persistence and T-cell immunosenescence in people aged fifty and older: a systematic review. Exp Gerontol. (2016) 77:87–95. 10.1016/j.exger.2016.02.005 26883338

[26] Geginat J Lanzavecchia A Sallusto F Proliferation and differentiation potential of human CD8+ memory T-cell subsets in response to antigen or homeostatic cytokines. Blood. (2003) 101:4260–6. 10.1182/blood-2002-11-3577 12576317

[27] Pittet MJ Valmori D Dunbar PR Speiser DE Liénard D Lejeune F. High frequencies of naive Melan-A/MART-1-specific CD8(+) T cells in a large proportion of human histocompatibility leukocyte antigen (HLA)-A2 individuals. J Exp Med. (1999) 190:705–15. 10.1084/jem.190.5.705 10477554 PMC2195613

[28] Voelter V Rufer N Reynard S Greub G Brookes R Guillaume P. Characterization of Melan-A reactive memory CD8+ T cells in a healthy donor. Int Immunol. (2008) 20:1087–96. 10.1093/intimm/dxn066 18573812

[29] Bhardwaj V Kumar V Geysen HM Sercarz EE. Degenerate recognition of a dissimilar antigenic peptide by myelin basic protein-reactive T cells. Implications for thymic education and autoimmunity. J Immunol. (1993) 151:5000–10. 7691962

[30] Hagerty DT Allen PM. Intramolecular mimicry. Identification and analysis of two cross-reactive T cell epitopes within a single protein. J Immunol. (1995) 155:2993–3001. 7673717

[31] Loftus DJ Castelli C Clay TM Squarcina P Marincola FM Nishimura MI. Identification of epitope mimics recognized by CTL reactive to the melanoma/melanocyte-derived peptide MART-1(27-35). J Exp Med. (1996) 184:647–57. 10.1084/jem.184.2.647 8760818 PMC2192745

[32] Grogan JL Kramer A Nogai A Dong L Ohde M Schneider-Mergener J. Cross-reactivity of myelin basic protein-specific T cells with multiple microbial peptides: experimental autoimmune encephalomyelitis induction in TCR transgenic mice. J Immunol. (1999) 163:3764–70. 10490973

[33] Hemmer B Fleckenstein BT Vergelli M Jung G McFarland H Martin R. Identification of high potency microbial and self ligands for a human autoreactive class II-restricted T cell clone. J Exp Med. (1997) 185:1651–9. 10.1084/jem.185.9.1651 9151902 PMC2196302

[34] Misko IS Cross SM Khanna R Elliott SL Schmidt C Pye SJ. Crossreactive recognition of viral, self, and bacterial peptide ligands by human class I-restricted cytotoxic T lymphocyte clonotypes: implications for molecular mimicry in autoimmune disease. Proc Natl Acad Sci USA. (1999) 96:2279–84. 10.1073/pnas.96.5.2279 10051632 PMC26774

[35] Brehm MA Pinto AK Daniels KA Schneck JP Welsh RM Selin. T cell immunodominance and maintenance of memory regulated by unexpectedly cross-reactive pathogens. Nat Immunol. (2002) 3:627–34. 10.1038/ni806 12055626

[36] Barnes E Ward SM Kasprowicz VO Dusheiko G Klenerman P Lucas M. Ultra-sensitive class I tetramer analysis reveals previously undetectable populations of antiviral CD8+ T cells. Eur J Immunol. (2004) 34:1570–7. 10.1002/eji.200424898 15162426

[37] Lima MA Marzocchetti A Autissier P Tompkins T Chen Y Gordon J. Frequency and phenotype of JC virus-specific CD8+ T lymphocytes in the peripheral blood of patients with progressive multifocal leukoencephalopathy. J Virol. (2007) 81:3361–8. 10.1128/JVI.01809-06 17229701 PMC1866063

[38] Alanio C Nicoli F Sultanik P Flecken T Perot B Duffy D. Bystander hyperactivation of preimmune CD8+ T cells in chronic HCV patients. ELife. (2015) 4:e07916. 10.7554/eLife.07916 26568315 PMC4752008

[39] Hui DJ Edmonson SC Podsakoff GM Pien GC Ivanciu L Camire RM. AAV capsid CD8+ T-cell epitopes are highly conserved across AAV serotypes. Mol Ther Methods Clin Dev. (2015) 2:15029. 10.1038/mtm.2015.29 26445723 PMC4588448

[40] Mingozzi F High KA. Immune responses to AAV vectors: overcoming barriers to successful gene therapy. Blood. (2013) 122:23–36. 10.1182/blood-2013-01-306647 23596044 PMC3701904

[41] D'Asaro M Dieli F Caccamo N Musso M Porretto F Salerno A. Increase of CCR7- CD45RA+ CD8 T cells (T(EMRA)) in chronic graft-versus-host disease. Leukemia. (2006) 20:545–7. 10.1038/sj.leu.2404079 16408100

[42] Barbarash L Kudryavtsev I Rutkovskaya N Golovkin A. T cell response in patients with implanted biological and mechanical prosthetic heart valves. Mediators Inflamm. (2016) 2016:1937564. 10.1155/2016/1937564 26989331 PMC4773556

[43] Erles K Sebökovà P Schlehofer JR. Update on the prevalence of serum antibodies (IgG and IgM) to adeno-associated virus (AAV). J Med Virol. (1999) 59:406–11. 10.1002/(SICI)1096-9071(199911)59:3<406::AID-JMV22>3.0.CO;2-N 10502275

[44] Murphy SL Li H Mingozzi F Sabatino D Hui D Edmonson S. Diverse IgG subclass responses to adeno-associated virus infection and vector administration. J Med Virol. (2009) 81:65–74. 10.1002/jmv.21360 19031458 PMC2782623

[45] Hernandez YJ Wang J Kearns WG Loiler S Poirier A Flotte TR Latent adeno-associated virus infection elicits humoral but not cell-mediated immune responses in a nonhuman primate model. J Virol. (1999) 73:8549–58. 10482608 10.1128/jvi.73.10.8549-8558.1999 PMC112875

[46] Salvetti A Orève S Chadeuf G Favre D Cherel Y Champion-Arnaud P. Factors influencing recombinant adeno-associated virus production. Human Gene Therapy. (1998) 9:695–706. 10.1089/hum.1998.9.5-695 9551617

[47] Schnepp BC Jensen RL Clark KR Johnson PR. Infectious molecular clones of adeno-associated virus isolated directly from human tissues. J Virol. (2009) 83:1456–64. 10.1128/JVI.01686-08 19019948 PMC2620885

[48] Gao G Vandenberghe LH Alvira MR Lu Y Calcedo R Zhou X. Clades of adeno-associated viruses are widely disseminated in human tissues. J Virol. (2004) 78:6381–8. 10.1128/JVI.78.12.6381-6388.2004 15163731 PMC416542

[49] Schnepp BC Jensen RL Chen C-L Johnson PR Clark KR. Characterization of adeno-associated virus genomes isolated from human tissues. J Virol. (2005) 79:14793–803. 10.1128/JVI.79.23.14793-14803.2005 16282479 PMC1287572

[50] Rohde V Erles K Sattler HP Derouet H Wullich B Schlehofer JR. Detection of adeno-associated virus in human semen: does viral infection play a role in the pathogenesis of male infertility? Fertil Steril. (1999) 72:814–6. 10.1016/S0015-0282(99)00363-5 10560983

[51] Hüser D Khalid D Lutter T Hammer E-M Weger S Heßler M High prevalence of infectious adeno-associated virus (AAV) in human peripheral blood mononuclear cells indicative of T lymphocytes as sites of AAV persistence. J Virol. (2016) 941:JVI02137-16. 10.1128/JVI.02137-16 PMC5286889 27928011

[52] Hussain T Quinn KM. Similar but different: virtual memory CD8 T cells as a memory-like cell population. Immunol Cell Biol. (2019) 97:675–84. 10.1111/imcb.12277 31140625

[53] Jacomet F Cayssials E Basbous S Levescot A Piccirilli N Desmier D. Evidence for eomesodermin-expressing innate-like CD8(+) KIR/NKG2A(+) T cells in human adults and cord blood samples. Eur J Immunol. (2015) 45:1926–33. 10.1002/eji.201545539 25903796

[54] Boutin S Monteilhet V Veron P Leborgne C Benveniste O Montus MF. Prevalence of serum IgG and neutralizing factors against adeno-associated virus (AAV) types 1, 2, 5, 6, 8, and 9 in the healthy population: implications for gene therapy using AAV vectors. Human Gene Therapy. (2010) 21:704–12. 10.1089/hum.2009.182 20095819

[55] Stanford S Pink R Creagh D Clark A Lowe G Curry N. Adenovirus-associated antibodies in UK cohort of hemophilia patients: a seroprevalence study of the presence of adenovirus-associated virus vector-serotypes AAV5 and AAV8 neutralizing activity and antibodies in patients with hemophilia A. Res Pract Thromb Haemost. (2019) 3:261–7. 10.1002/rth2.12177 31011710 PMC6462753

[56] Kuranda K Jean-Alphonse P Leborgne C Hardet R Collaud F Marmier S. Exposure to wild-type AAV drives distinct capsid immunity profiles in humans. J Clin Invest. (2018) 128:5267–79. 10.1172/JCI122372 30352429 PMC6264647

[57] Northfield JW Loo CP Barbour JD Spotts G Hecht FM Klenerman P. Human immunodeficiency virus type 1 (HIV-1)-specific CD8+ T(EMRA) cells in early infection are linked to control of HIV-1 viremia and predict the subsequent viral load set point. J Virol. (2007) 81:5759–65. 10.1128/JVI.00045-07 17376902 PMC1900265

[58] Vandamme C Adjali O Mingozzi F. Unraveling the complex story of immune responses to AAV vectors trial after trial. Hum Gene Ther. (2017) 28:1061–74. 10.1089/hum.2017.150 28835127 PMC5649404

