# Supplementary material for: Tetramer-Based Enrichment of Preexisting Anti-AAV8 CD8+ T Cells in Human Donors Allows the Detection of a TEMRA Subpopulation
Source: Front Immunol. 2020 Jan 21;10:3110. doi: 10.3389/fimmu.2019.03110 (PMC6990124; doi:10.3389/fimmu.2019.03110)
Supplement: Supplementary file 1 [file Data_Sheet_1.PDF]

Supplementary Material

1.1 Anti-AAV ELISA

Detection of anti-AAV2 and anti-AAV8 total IgG antibodies in the plasma of healthy human donors was conducted using an enzyme-linked immunosorbent assay (ELISA). Nunc Maxisorp P96 plates (Sigma-Aldrich) were coated overnight at 4°C with rAAV2 or rAAV8 particles (INSERM UMR 1089 Vector Core Facility). After washing and plate saturation, wells were then incubated for 1 hour at 37°C with 12 serial twofold dilutions of human plasma (from 1/10 to 1/20,480) followed by HRP-conjugated anti-human $F(ab')_2$ IgG antibody (Jackson) for 1 hour at 37°C. Positive controls consisted in human sera previously identified to contain anti-AAV2 or anti-AAV8 antibodies, as well as in anti-AAV antibodies (Progen). Revelation was performed using 3,3'-5,5'-Tetramethylbenzidine (TMB, OptEIA, BD Biosciences) and absorbance of duplicate samples was read at 450 nm with a correction at 570 nm on a MultiSkan Go reader (Thermo Scientific). Positive threshold curves for each ELISA were determined from seronegative human sera (> 10 donors) as the mean optic density for each dilution + 2*SD. For each donor, anti-AAV2 or anti-AAV8 IgG titer was defined as the last plasma dilution with an optic density remaining above the threshold curve.

1.2 Anti-AAV Neutralization Assay

Detection of anti-AAV8 and anti-AAV2 neutralizing factors in the decomplemented plasma of healthy human donors was conducted using a neutralization assay based on the principle of a transduction inhibition assay. Briefly, 10-fold serial dilutions of plasma (1:10 to 1:100,000) were incubated 30 min at room temperature with rAAV8-CMV-LacZ or rAAV2-CMV-LacZ vectors and added to the cells at a MOI of 4,000 vg/cell. After a 24H-incubation at 37°C, cells were fixed and β-galactosidase expression was then revealed with an X-gal substrate (Promega). Titers of anti-AAV neutralizing factors were defined as the last dilution were transduction was reduced compared to positive control wells, where cells were transduced with rAAV8 or rAAV2 vectors in the absence of plasma.

Supplementary Figures

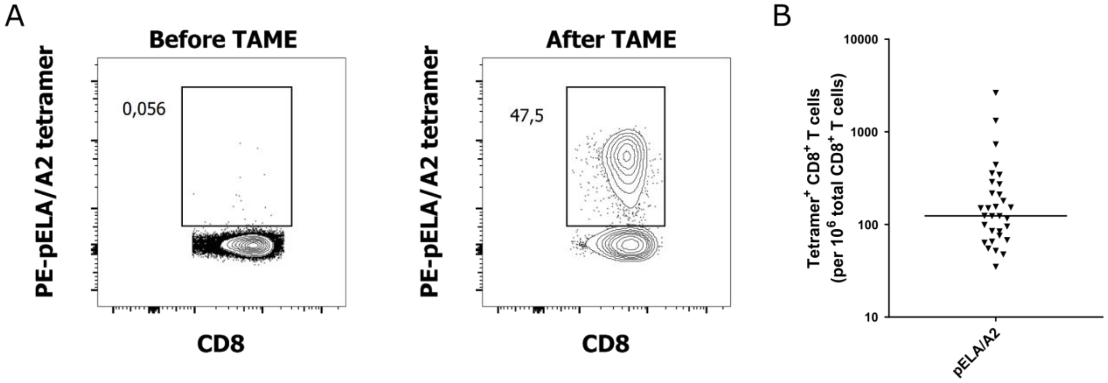

Figure S1. Detection of Melan-A-specific $CD8^+$ T lymphocytes in PBMCs from $A2^+$ donors. (A) Representative dot plots obtained before and after tetramer enrichment with pELA/A2 tetramer complexes. Percentages of PE-$Tetramer^+$ cells among total $CD8^+$ T cells obtained before and after tetramer enrichment are indicated. PE: phycoerythrin. **(B)** *Ex vivo* frequencies of pELA/A2 $Tetamer^+$ $CD8^+$ T lymphocytes in $A2^+$ donors. *Ex* vivo frequencies of Melan-A-specific $CD8^+$ T cells were calculated by dividing the absolute number of PE-$Tetramer^+$ $CD8^+$ T cells, detected after tetramer enrichment, by the absolute number of total $CD8^+$ T cells. The horizontal bar refers to the median value. Each symbol represents an individual donor (n=31).

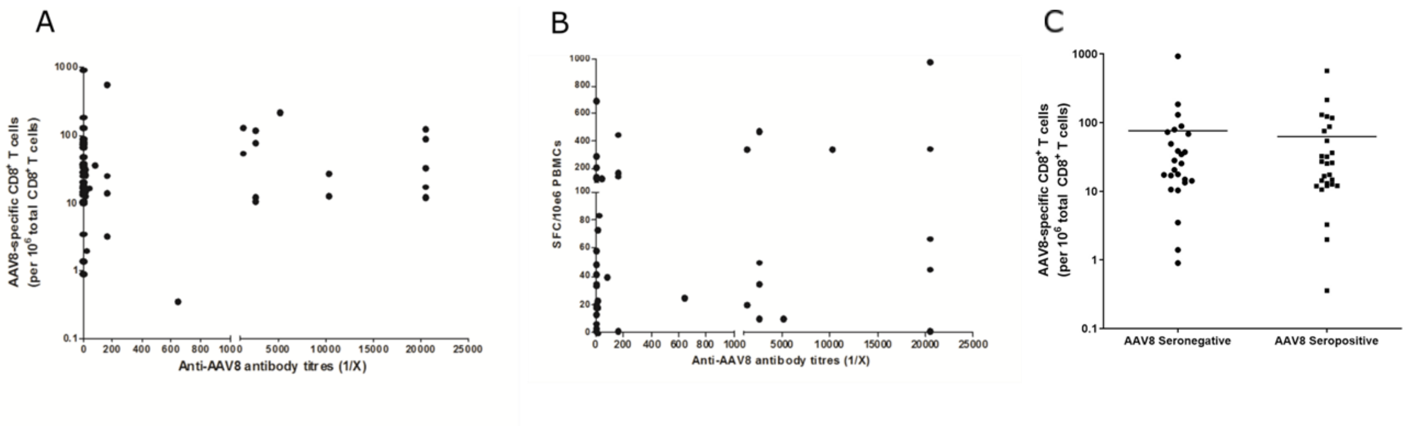

Figure S2. Correlation study between humoral and cellular responses. (A) Correlation study between *ex vivo* frequencies of AAV8-specific $CD8^+$ T cells and anti-AAV8 antibody titers. *Ex vivo* frequencies of AAV8-specific $CD8^+$ T cells, determined after tetramer enrichment, were plotted against titers of anti-AAV8 total IgG antibodies, determined through ELISA. Spearman correlation test revealed no correlation between both parameters. **(B)** Correlation study between anti-AAV8 IFNγ ELISpot responses and anti-AAV8 antibody titers. SFC/10^{e}6 cells values obtained through anti-AAV8 IFNγ ELISpot assays were plotted against titers of anti-AAV8 total IgG antibodies, determined by ELISA. Spearman correlation test revealed no correlation between both parameters. **(C)** Detection of AAV8-specific $CD8^+$ T lymphocytes in AAV8 seropositive and seronegative A2+ donors. Ex vivo frequencies of AAV8-specific $CD8^+$ T cells were calculated by dividing the absolute number of PE-enriched $Tetramer^+$ $CD8^+$ T cells by the absolute number of total $CD8^+$ T cells. Results are represented as the number of $tetramers^+$ $CD8^+$ T cells per 10^6 total $CD8^+$ T cells for anti-AAV8 IgG antibody negative donors (AAV8 Seronegative) or positive donors (AAV8 Seropositive). Horizontal bars refer to median values. Each symbol represents an individual.

Supplementary Tables

Table S1. Sequences of peptides selected to construct pAAV2/A2, pAAV8/A2 and pAAV8/B7 tetramer complexes.

**pAAV2/A2**<br>**(A2-restricted AAV2 peptides[a])**	**pAAV8/A2**<br>**(A2-restricted AAV8 peptides[a])**	**pAAV8/B7**<br>**(B7-restricted AAV8 peptides[a,b])**
FMVPQYGYLTL	FMIPQYGYLTL	IPQYGYLTL[b]
RLMNPLIDQYL	FMIPQYGYL	RPKRLSFKL
LMNPLIDQYL	YLPDWLEDNL	SPLMGGFGL
YLQGPIWAKI	RLSFKLFNIQV	VPADPPTTF
LIDQYLYYL	LQAGDNPYL	QPARKRLNF
KLFNIQVKEV	FQFTYTFEDV	FPSNGILIF
FTFSYTFEDV	GMVWQNRDVYL	APSGVGPNTM
RLNFKLFNI	FMIPQYGYLT	SPRDWQRLI
QLDSGDNPYL	ILIKNTPVPA	LPTYNNHL
GMVWQDRDVYL		APGKKRPV
RQLDSGDNPYL		RPIGTRYLTRNL
SLDRLMNPL		RPIGTRYLT
RVLEPLGLV		
**Total = 13 peptides**	**Total = 9 peptides**	**Total = 12 peptides**

[a] For each case, candidate epitopes were first issued after interrogation of bioinformatics prediction databases (SYFPEITHI epitope prediction, NetMHC 3.2 Server, IEDB MHC I Processing Cleavage Prediction). The first 30 epitopes were then screened through UV-mediated peptide exchange[1].

Selected epitopes were then used to construct tetramer complexes. For each case, tetramer complexes were used as a single pool to stain PBMCs.

[b] Using the method described above, the B7-restricted AAV8 immunodominant peptide originally described by Mingozzi *et al*. was selected[2].

Table S2. Antibodies used for cytometric analysis.

Antibody	Supplier	Reference	Clone
CD3 PerCP Cy5.5	BD Biosciences	560835	UCHT1
CD4 APC-H7	BD Biosciences	560251	RPA-T4
CD8 eFluor 605	eBiosciences	93-0088-42	
CD14 AF700	BD Biosciences	561029	M5E2
CD16 AF700	BD Biosciences	557920	3G8
CD19 AF700	BD Biosciences	561031	HIB19
CD45RO BV421	BD Biosciences	562641	UCHL1
CD27 PeCy7	BD Biosciences	560609	M-T271
CD45RA PE-Dazzle 594	BioLegend	304146	HI100
CCR7 (CD197) BV785	BioLegend	353229	G043H7
FVD eFluor 506	eBiosciences	65-0866-14	-

Table S3: Overview of anti-AAV8 and anti-AAV2 humoral responses assessed on plasma from $A2^+$ and $B7^+$ donors.

	Anti-AAV8 total IgG antibodies[a]		Anti-AAV8 neutralizing factors[b]
**HLA Allele**	**Donors tested**	**Donors with titres ≥ 1/10**	**Donors with titres ≥ 1/50**
**$A2^+/B7^-$**	37	20 (54.1 %)	16 (43.2 %)
**$A2^-/B7^+$**	14	8 (57.1 %)	9 (64.3 %)
**$A2^+/B7^+$**	7	2 (28.6 %)	4 (57.1 %)
**Total**	**58**	**30 (51.7 %)**	**29 (50 %)**
**Median Titres**		**1:2560**	**1:1000**

[a]Anti-AAV8 total IgG antibodies were assessed through ELISA.

[b]Anti-AAV8 neutralizing factors were assessed through neutralization assay.

Supplementary references

1. Rodenko, B. *et al.* Generation of peptide-MHC class I complexes through UV-mediated ligand exchange. *Nat. Protoc.* **1,** 1120–1132 (2006).

2. Mingozzi, F. *et al.* CD8+ T-cell responses to adeno-associated virus capsid in humans. *Nat. Med.* **13,** 419–422 (2007).
